# Editorial: Nutritional and Physical Activity Strategies to Boost Immunity, Antioxidant Status and Health

**DOI:** 10.3389/fphys.2022.846261

**Published:** 2022-03-18

**Authors:** Mallikarjuna Korivi, Veeranjaneya Reddy Lebaka, Arifullah Mohammed, Weibing Ye

**Affiliations:** ^1^Exercise and Metabolism Research Center, College of Physical Education and Health Sciences, Zhejiang Normal University, Jinhua, China; ^2^Department of Microbiology, Yogi Vemana University, Kadapa, India; ^3^Faculty of Agro-Based Industry and Institute of Food Security and Sustainable Agriculture, Universiti Malaysia Kelantan, Jeli, Malaysia

**Keywords:** dietary intake, inflammation, oxidative stress—related enzymes, exercise, physical health

## Summary

In this thematic collection, we intended to explore the role of nutritional supplements and physical activity on various forms of immunity, inflammatory response, redox signaling, and health. A study demonstrated that supplementation of Santé premium silver perch essence (SPSPE) delayed swimming fatigue and attenuated exhaustive swimming exercise-induced lipid peroxidation and myoglobin induction in rats (Chen C.-Y. et al.). The SPSPE is rich in proteins, collagen, trace elements, minerals, and branch chain amino acid (Chen C.-Y. et al.). Another study reported that “turtle oil,” which is extracted from the fat of Chinese soft-shelled turtle (*Pelodiscus sinensis*), comprised a highest percentage of unsaturated fatty acids (UFAs), including ogema-3 poly UFAs (~22%), and omega-9 mono-UFAs (~30%) (Yang et al.). Feeding of this turtle oil to aging rats in combination with swimming exercise improved spatial memory, physical strength, antioxidant status (superoxide dismutase) and maintained stable blood pressure in aging rats (Yang et al.). The beneficial effect of combined intervention was further emphasized by Chen C.-N. et al. in middle-aged adults with obesity. In this randomized controlled trial, combination of high-protein diet and exercise intervention (12-week) resulted a significant decrease of fat mass and lipid profiles, and improvement of insulin sensitivity, glucose tolerance and inflammation, which indicates improved cardiometabolic health (Chen C.-N. et al.). Dietary intake of micronutrients (calcium, vitamin D, zinc, and selenium) also contributes to promote cardiovascular health (Narayanam et al.). However, due to rapid changes in lifestyle, people couldn't intake sufficient dietary nutrients that result in huge dependency on dietary supplements. Whilst, supplements can cause sudden rise in circulating micronutrients which may cause cardiovascular damage (Narayanam et al.). Therefore, dietary intake of sufficient nutrients with or without combination of physical activity could boost immunity for all ages of adults with or without metabolic disorders.

Conversely, insufficient intake of certain nutrients (iron, vitamins A, B12, D, E, folate, and copper) lead to low concentrations of hemoglobin, known as “nutritional anemia.” A study on Malaysian men reported that body mass index (BMI) of individual is a potent anthropometric index to predict the anemia (Dutta et al.). Besides, malnutrition is closely associated with the incidence of tuberculosis (TB), and treatment outcomes affected by protein calorie malnutrition in patients. Usually, pathogenesis of lung injury in TB patients is depends on persons' immune system and/or healthy bodyweight. A study claimed that BMI and total serum protein levels of TB drug addicts were normal, but hemoglobin and albumin levels were significantly lower compared with non-TB drug addicts (Jia et al.). These findings emphasize the importance of nutrition in disease treatment and prevention. An interesting animal study demonstrated that inflammatory lung injury induced by particulate matter 2.5 (PM_2.5_) exposures was effectively attenuated by aerobic exercise training (Qin et al.). This was evidenced by alleviated airway obstruction, ultrastructural damage and inflammatory response in exercised rats against PM_2.5_ exposure (Qin et al.). Furthermore, a meta-analysis concluded that exercise intervention together with calorie restriction improved inflammatory response in overweight and obese adults (Liu et al.). To be particular, the decreased C-reactive protein (CRP), interleukin-6 (IL-6), and tumor necrosis factor-α (TNF-α) levels with combination intervention was effective in overweight and obese adults who had active lifestyle (Liu et al.). Taken together, our Research Topic summarizes that the beneficial effects are greater with combination of nutritional supplements and exercise than either type of intervention alone, in improving the inflammatory response, immunity, and antioxidant status.

## Author Contributions

MK and VRL drafted, edited, and finalized the editorial. AM and WY organized the articles sequence and extracted essential information. All authors contributed to the article and approved the submitted version.

## Funding

This study was partially supported by the Zhejiang Provincial Natural Science Foundation of China (LGF19H160021).

## Conflict of Interest

The authors declare that the research was conducted in the absence of any commercial or financial relationships that could be construed as a potential conflict of interest.

## Publisher's Note

All claims expressed in this article are solely those of the authors and do not necessarily represent those of their affiliated organizations, or those of the publisher, the editors and the reviewers. Any product that may be evaluated in this article, or claim that may be made by its manufacturer, is not guaranteed or endorsed by the publisher.

